# Effects of carnosine on the embryonic development and TiO_2_ nanoparticles-induced oxidative stress on Zebrafish

**DOI:** 10.3389/fvets.2023.1148766

**Published:** 2023-03-23

**Authors:** Giuseppe Caruso, Elena Maria Scalisi, Roberta Pecoraro, Vincenzo Cardaci, Anna Privitera, Emanuela Truglio, Fabiano Capparucci, Romana Jarosova, Antonio Salvaggio, Filippo Caraci, Maria Violetta Brundo

**Affiliations:** ^1^Department of Drug and Health Sciences, University of Catania, Catania, Italy; ^2^Unit of Neuropharmacology and Translational Neurosciences, Oasi Research Institute-IRCCS, Troina, Italy; ^3^Department of Biological, Geological and Environmental Sciences, University of Catania, Catania, Italy; ^4^Vita-Salute San Raffaele University, Milan, Italy; ^5^Scuola Superiore di Catania, University of Catania, Catania, Italy; ^6^Department of Biomedical and Biotechnological Sciences, University of Catania, Catania, Italy; ^7^Department of Chemical, Biological, Pharmaceutical and Environmental Sciences, University of Messina, Messina, Italy; ^8^Department of Chemistry and R.N. Adams Institute for Bioanalytical Chemistry, University of Kansas, Lawrence, KS, United States; ^9^Experimental Zooprophylactic Institute of Sicily, Palermo, Italy

**Keywords:** carnosine, oxidative stress, reactive oxygen species, heat shock proteins, metallothioneins, zebrafish

## Abstract

Oxidative stress is due to an unbalance between pro-oxidants, such as reactive oxygen (ROS) and nitrogen (RNS) species, and antioxidants/antioxidant system. Under physiological conditions these species are involved in different cellular processes such as cellular homeostasis and immune response, while an excessive production of ROS/RNS has been linked to the development of various diseases such as cancer, diabetes, and Alzheimer's disease. In this context, the naturally occurring dipeptide carnosine has shown the ability to scavenge ROS, counteract lipid peroxidation, and inhibit proteins oxidation. Titanium dioxide nanoparticles (TiO_2_-NPs) have been widely used to produce cosmetics, in wastewater treatment, in food industry, and in healthcare product. As consequence, these NPs are often released into aquatic environments. The *Danio rerio* (commonly called zebrafish) embryos exposure to TiO_2_-NPs did not affect the hatching rate, but induced oxidative stress. According to this scenario, in the present study, we first investigated the effects of carnosine exposure and of a sub-toxic administration of TiO_2_-NPs on the development and survival of zebrafish embryos/larvae measured through the acute embryo toxicity test (FET-Test). Zebrafish larvae represent a useful model to study oxidative stress-linked disorders and to test antioxidant molecules, while carnosine was selected based on its well-known multimodal mechanism of action that includes a strong antioxidant activity. Once the basal effects of carnosine were assessed, we then evaluated its effects on TiO_2_-NPs-induced oxidative stress in zebrafish larvae, measured in terms of total ROS production (measured with 2,7-dichlorodihydrofluorescein diacetate probe) and protein expression by immunohistochemistry of two cellular stress markers, 70 kDa-heat shock protein (Hsp70) and metallothioneins (MTs). We demonstrated that carnosine did not alter the phenotypes of both embryos and larvae of zebrafish at different hours post fertilization. Carnosine was instead able to significantly decrease the enhancement of ROS levels in zebrafish larvae exposed to TiO_2_-NPs and its antioxidant effect was paralleled by the rescue of the protein expression levels of Hsp70 and MTs. Our results suggest a therapeutic potential of carnosine as a new pharmacological tool in the context of pathologies characterized by oxidative stress such as neurodegenerative disorders.

## 1. Introduction

Carnosine is a naturally occurring peptide composed of β-alanine and L-histidine and synthesized through an ATP-dependent reaction by carnosine synthetase 1 (CARNS1) enzyme. This peptide can be found at very high concentrations in mammalian tissues, particularly in the brain, where it reaches concentrations ranging between 0.7 and 2.0 mM (up to 5 mM in some areas), as well as in cardiac and skeletal muscles (up to 20 mM), and also in the tissues of some species of invertebrates (1, 2). Different studies have also shown the presence of carnosine in numerous other classes of Vertebrates including fish (3, 4), while plants do not contain it at all (5).

Many beneficial activities have been attributed to carnosine, such as pH-buffering activity (6), heavy metal chelating activity (7), antioxidant, anti-glycating, and ion-chelating capacity (8), along with the ability to scavenge free-radicals (9). When the synthesis of reactive and pro-oxidant species overtakes the antioxidant defense system, oxidative stress occurs (10, 11). In this context, carnosine has shown the ability to scavenge reactive oxygen species (ROS) and alpha–beta-unsaturated aldehydes formed from peroxidation of cell membrane fatty acids during oxidative stress (12), also inhibiting the oxidative modifications of proteins exposed to hydroxyl radicals (13, 14).

The over-production of ROS and the related oxidative stress have been shown to disrupt cellular proteins (15), requiring their subsequent refolding; in this context, the activity exerted by heat shock proteins (HSPs) represents the most effectively protective mechanism (16). HSPs are a group of molecular chaperones able to revert or inhibit denaturation or unfolding of cellular proteins in response to stress and/or high temperature. Recent studies have been focused on the regulatory role of HSPs in several redox processes as well as on their protection of antioxidant mediators (17). In particular, the 70 kDa-HSP (Hsp70) family promotes the proteolytic removal of oxidatively damaged proteins by the proteasome (18) and closely interact with the nitric oxide generation systems (19). Hsp70 is represents a marker of oxidative response in different experimental models, including *Danio rerio* (commonly called zebrafish) (20–22).

Metallothioneins (MTs) are a group of low molecular weight metal-binding proteins with high affinity for divalent metal ions. MTs have been widely considered for their protective role that is mediated by their ability to exert metal detoxification (23) and counteract oxidative stress-induced damage (24).

Among xenobiotic, nanoparticles (NPs) could have a significant role in the development of oxidative stress phenomena owing to their small surface-area-to-volume ratio, physical size, and relatively high biopersistence. In particular, metal oxide NPs lead to ROS generation directly or indirectly, with an extent that depends on dose, metal speciation, and exposure route (25). The NPs-induced ROS formation, and in general ROS over-production, has been linked to lipid and protein peroxidation as well as to DNA fragmentation and reduced antioxidant ability (26, 27). Titanium dioxide NPs (TiO_2−_NPs) are widely used to produce cosmetics, wastewater treatment, food, and healthcare products. It has been demonstrated that exposure to TiO_2_-NPs does not affect the hatching rate of zebrafish embryos, and does not cause malformation on the larvae (28), even if it can lead to oxidative stress in embryos (29). Thus, considering the oxidative capacity of TiO_2_-NPs and the multimodal mechanism of action of carnosine (30), including the well-known antioxidant activity also demonstrated in zebrafish embryos (31), we investigated whether carnosine could inhibit the oxidative stress induced by TiO_2_-NPs in this model.

*Z*ebrafish is a teleost fish widely used in translational research (32), allowing to investigate the molecular mechanisms related to numerous diseases including neurodegenerative and immune diseases (33–36); in particular, this animal model is considered of utmost importance in the case of (eco)toxicological studies. Zebrafish possesses several advantages over rodent models in the study of vertebrate development and disease, including high fecundity rate (37, 38). Zebrafish eggs are transparent, allowing the observation of morphogenetic changes and organogenesis in real time (39). In particular, embryos have been shown to represent a valuable tool to study both pro-oxidant (40) and pro-inflammatory (41) phenomena, as well as to investigate the therapeutic potential of nutraceuticals (42) and natural products (43). Additionally, embryos have rapid development with embryogenesis being complete by 72 hours post fertilization (hpf) and most organs fully developed by 96 hpf.

Zebrafish model represents the perfect bridge in preclinical toxicology between *in vitro* assays and mammalian *in vivo* studies (44). It has also been established as a valuable animal model to study oxidative stress phenomena in different diseases, including diabetic retinopathy and nephropathy (45, 46). In this context, carnosine treatment has shown to be able to rescue pancreas disruption (31) and microvascular alterations (46). With regard to zebrafish development, carnosine has been linked to olfactory and visual function (47), while the effects of carnosine exposure during zebrafish embryonic development have not yet been completely elucidated.

In the present study, we first investigated the effects of carnosine exposure and of a sub-toxic administration of TiO_2_-NPs on the development and survival of zebrafish embryos/larvae. Once the safety of carnosine was assessed, we investigated its effects on the TiO_2_-NPs-induced oxidative stress in zebrafish, measured in terms of total ROS production and protein expression of two well-known markers of cellular stress, Hsp70 and MTs.

## 2. Materials and methods

### 2.1. Materials and reagents

All chemicals were of analytical grade and purchased from Sigma-Aldrich (St. Louis, MO, USA) or Thermo Fisher Scientific Inc. (Pittsburgh, PA, USA) unless specified otherwise.

### 2.2. Preparation of work solutions

A stock solution of carnosine at the concentration of 1 M was prepared by dissolving the powder in osmosis water, which is optimal for housing zebrafish (48). Working solutions of 0.1, 1, 10, and 20 mM were prepared by diluting the stock solution in osmosis water. TiO_2_-NPs, kindly supplied by the CNR-IMM of Catania, characterized by a crystalline phase mix of anatase (86%) and rutile (14%), and with an average size of about 50 nm, were diluted to 0.1 mg/mL (working solution) in osmosis water. In order to avoid the formation of NP aggregates, it was necessary to carry out 4 cycles of 10 min (with 3 min of break) of sonication by using a probe sonicator (Sonopuls, De Marco S.r.l., Milan, Italy), with a frequency of 40 kHz (49).

### 2.3. Acute toxicity experiment of zebrafish embryos

Fertilized eggs from the same spawning event and collected within 4 hpf were provided by the Fish Pathology and Experimental Center of Sicily (CISS) of the Department of Veterinary Science (University of Messina). Only eggs at the blastula stage were used, while the infertile eggs or eggs with alterations on the chorion were discarded. According to OECD Test Guidelines for Chemicals (50) we used the 24-well plates to distribute the eggs (1 embryo/well, 2 mL solution/well).

The first set of experiments was carried out to investigate the effects of increasing concentrations of carnosine (0.1, 1, 10, and 20 mM) as well as of TiO_2_-NPs (0.1 mg/mL) on zebrafish embryonic development. We set up 24-well plates for each concentration of carnosine selected and TiO_2_-NPs (0.1 mg/mL). We performed three replicates for each multi-well plates prepared.

In particular, in all 24-well plates set up, 20 embryos were exposed to test concentration and 4 embryos were exposed to dilution water as internal plate control. A total number of 60 embryos were used for each experimental condition. As recommended by the protocol procedure, 24-well plates of positive controls and negative controls have been made; for the negative controls, embryos were exposed to water dilution, while in positive controls, embryos were exposed to 3,4-dichloroaniline (DCA) at the concentration of 0.004 mg/mL in water (51).

During the experiments, the temperature within the wells was maintained constant (26 ± 1°C) and the solution in each well was replaced every 24 h (semi-static renewal) (50).

Since neither carnosine nor TiO_2_-NPs induced toxic effects, we selected a starting concentration of 20 mM carnosine which represents the highest concentration of carnosine found at the tissue level. The 24-well plates of 20 mM carnosine solutions were prepared as a pre-treatment for embryos (1 h of pre-treatment), subsequently TiO_2_-NPs solution was added until the end of the test. Three replicates were performed, therefore a total number of 60 embryos was used.

A binocular microscope (E200 MV-R LED, Nikon Instruments S.p.A., Florence, Italy) equipped with a camera (CMOS, Nikon Instruments S.p.A.) was used to observe and photograph the embryos every 24 h (until the end of the test: 96 hpf).

The acute toxicological endpoints (coagulated embryos, lack of somite formation, non-detachment of the tail, and lack of heartbeat) were assessed and quantified as “observed” or “not observed”. A DanioScope™ software (Noldus Information Technology bv, Wageningen, Netherlands) was used to evaluate heartbeat, body length of larvae, and possible malformations.

According to OECD, the acute embryo toxicity test (FET-Test; Fish Embryo Toxicity-Test) was considered valid only if overall survival of embryos was ≥90% in the negative control (dilution-water) and ≤ 70% (minimum mortality of 30%) in the positive control (0.004 mg/mL of DCA for zebrafish) until the end of the 96 h exposure. After hatching, the larvae of each experimental group were used to evaluate intracellular ROS and protein expression of Hsp70 and MTs through immunohistochemical analysis.

### 2.4. Evaluation of intracellular ROS

2,7-dichlorodihydrofluorescein diacetate (H2DCFDA) was used to detect intracellular ROS content. At the end of the exposure, all larvae, including controls, were stained with a ROS detection solution. The ROS detection solution (5 μM) was prepared in Hanks' balanced salt solution (HBSS). Larvae were washed twice with HBSS for 2 min, then incubated with the ROS detection solution for a total of 15 min at 28°C. At the end of the incubation, the ROS detection solution was discarded, the larvae were washed with HBSS (twice for 2 min), and placed on a glass slide. As a last step, larvae were examined by using a fluorescence microscope (NIKON ECLIPSE Ci, Nikon Instruments S.p.A.), equipped with a NIKON DS-Qi2 camera (Nikon Instruments S.p.A.). Image J software was used to measure fluorescence signals (52).

### 2.5. Immunohistochemical analysis

Immunohistochemical analysis was performed to evaluate the expression of Hsp70 or MTs in the whole larvae as previously described by Pecoraro et al. (51). Briefly, the larvae collected from each experimental group were washed in phosphate buffered saline (PBS) and fixed with 4% (w/v) paraformaldehyde. Next, the fixative was discarded and larvae were washed twice in PBS. The permeabilization of larvae, to improve antibody penetration, was obtained by using a solution of PBS-Triton X-100 (for 15 min). A blocking solution containing bovine serum albumin was used to block non-specific antibody binding. Larvae were placed on a glass slide and incubated overnight at 4°C with primary antibodies: anti-HSP70 polyclonal antibody (GeneTex, 1:1,000; Cat. No. GTX112963) or anti-MTs (GeneTex, 1:1,000; Cat. No. GTX12228). Larvae were then washed twice with PBS-Tween 20 to remove the excess of primary antibodies and incubated for 1 h at 4°C in the dark with TRITC-conjugated anti-rabbit (1:1,000) or FITC-conjugated anti-mouse (1:1,000) secondary antibodies. Following a washing step in PBS-Tween 20, the larvae were dehydrated employing increasing alcoholic solutions (70°, 80°, and 95°) and air dried. The larvae were mounted with 4′,6-diamidino-2-phenylindole (DAPI) (Abcam, Cambridge, UK) and sealed with rubber cement to be examined with a fluorescence microscope (NIKON ECLIPSE Ci) equipped with a NIKON DS-Qi2 camera. TRITC-conjugated anti-rabbit secondary antibody exhibited a red fluorescence, while FITC-conjugated anti-mouse secondary antibody exhibited a green fluorescence. The images obtained by fluorescence microscope have been processed with Image J software (52).

### 2.6. Statistical analysis

Statistical analysis was performed by using Graphpad Prism software, version 8.0 (Graphpad software, San Diego, CA, USA). Data are always reported as the mean ± standard deviation (SD). One-way analysis of variance (ANOVA), followed by Tukey's *post hoc* test, was used for multiple comparisons. The assumptions for performing the parametric tests were confirmed by applying the Brown–Forsythe test. Only *p*-values of < 0.05 were considered statistically significant.

## 3. Results

### 3.1. Carnosine and TiO_2_-NPs do not alter the development of zebrafish larvae

**Figure 1:**
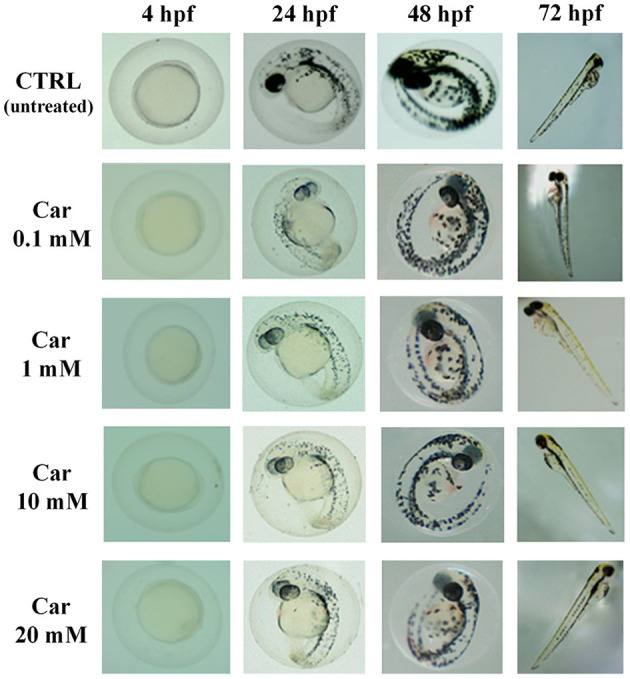
Effects of increasing concentrations of carnosine (0.1, 1, 10, and 20 mM) on the phenotypes of both embryos and larvae at different (4, 24, 48, and 72) hpf. Car, carnosine.

**Figure 2:**
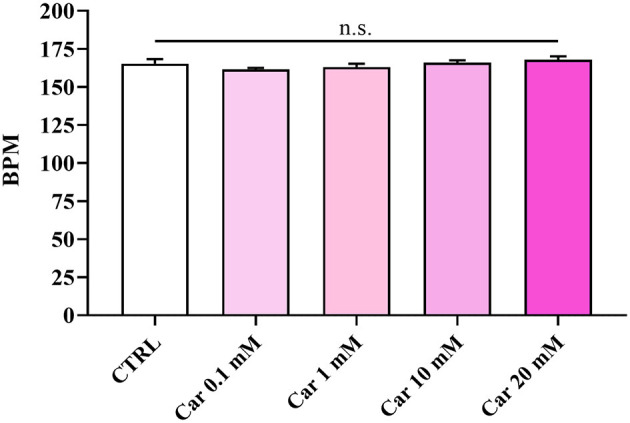
Beats per minute (BPM) of unexposed larvae and larvae exposed to increasing concentrations of carnosine (0.1, 1, 10, and 20 mM). SD is represented by vertical bars. Car, carnosine; n.s., not significant.

**Figure 3:**
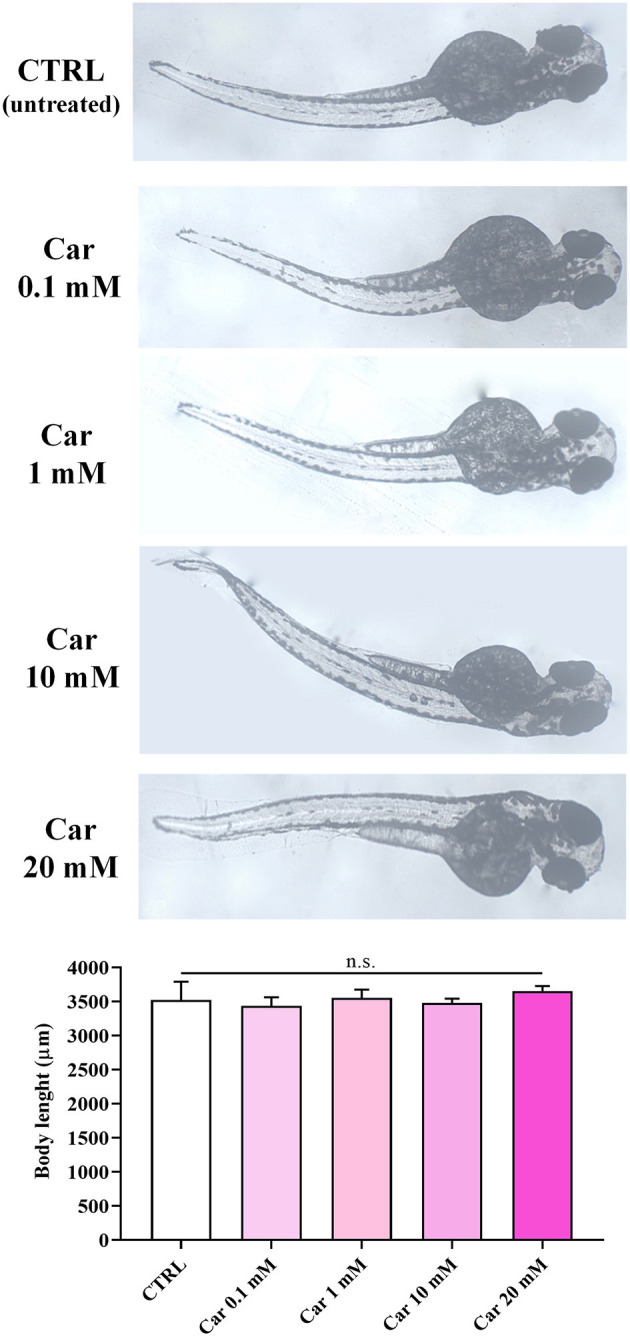
Body length (μm) of unexposed larvae and larvae exposed to increasing concentrations of carnosine (0.1, 1, 10, and 20 mM). SD is represented by vertical bars. Car, carnosine; n.s., not significant.

**Figure 4:**
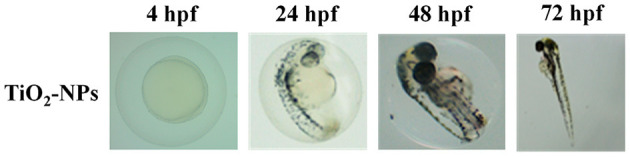
Effects of TiO_2_-NPs on the phenotypes of both embryos and larvae at different (4, 24, 48, and 72) hpf.

The first set of experiments was carried out to investigate the effects of increasing concentrations of carnosine (0.1, 1, 10, and 20 mM) on zebrafish embryonic development. As shown in Figure 1, none of the carnosine concentrations tested altered the development of zebrafish larvae compared to CTRL (untreated zebrafish larvae). We also performed a DanioScope™ analysis showing no alterations in the heartbeat of the embryos/larvae (Figure 2) as well as in the body length of the larvae (Figure 3). Beats per minute (BPM) values ranged from 161.7 to 167.9, with the highest values observed in embryos exposed to 20 mM carnosine. All the measured BPM values are to be considered physiological; in fact, in zebrafish the physiological heart rate is about 120–180 BPM (53, 54). With regard to the body length of the larvae, the measured values ranged from 3,433 to 3,653 μm. Once again, the highest values were observed in the case of embryos exposed to 20 mM carnosine. As in the case of BPM measurements, the body length of the larvae are to be considered physiological (39). As previously mentioned, it has been demonstrated that exposure to TiO_2_-NPs does not affect the hatching rate of embryos and does not cause malformation on the larvae, even if it can lead to oxidative stress in embryos. In order to confirm the absence of acute toxicity, the effect of TiO_2_-NPs treatment (0.1 mg/mL) on the phenotypes of both embryos and larvae at different hpf was also evaluated. As expected, the embryos completed their embryonic development, thus no toxicological endpoints have been recorded (Figure 4). The hatching rate was not altered, neither statistically significant differences in the survival rate were observed in the experimental groups compared to the control group. Hatching occurred 72 hpf in all experimental groups, and the larvae exhibited a good shape of body.

### 3.2. Carnosine decreases ROS production in TiO_2_-NPs-treated larvae

**Figure 5:**
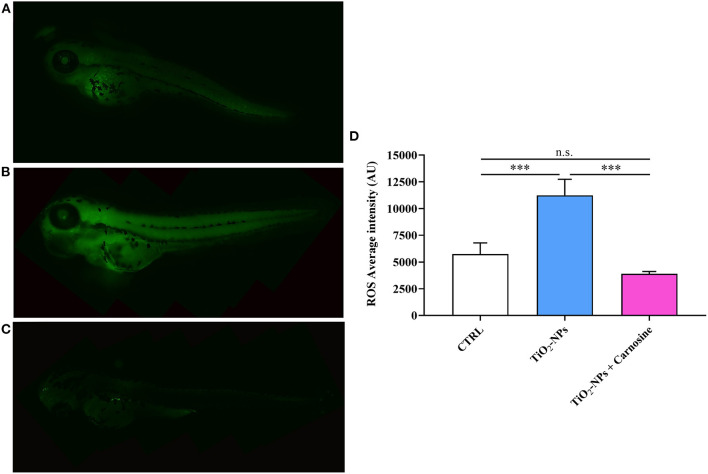
Total ROS production in **(A)** untreated larvae, **(B)** TiO_2_-NPs-treated larvae, and **(C)** TiO_2_-NPs-treated larvae in the presence of carnosine 20 mM (1 h pre-treatment). Carnosine was kept during the exposure to TiO_2_-NPs. The average fluorescence intensity (AU) of at least 5 values for fixed larva is reported in **(D)**. SD is represented by vertical bars. ***Significantly different, *p* < 0.001. n.s., not significant.

With regard to the evaluation of ROS production under our experimental conditions, it was found a significant increase of these reactive species in TiO_2_-NPs-treated larvae compared to CTRL (*p* < 0.001) (Figure 5). The pre-treatment with carnosine at the concentration of 20 mM was able to completely prevent the production of ROS compared to TiO_2_-NPs-treated larvae (*p* < 0.001), giving fluorescence values comparable to that observed in untreated larvae. Despite a trend of decrease observed in TiO_2_-NPs-treated larvae in the presence of carnosine compared to untreated larvae, the difference was not statistically significant.

### 3.3. Carnosine decreases Hsp70 and MTs oxidative stress response markers

**Figure 6:**
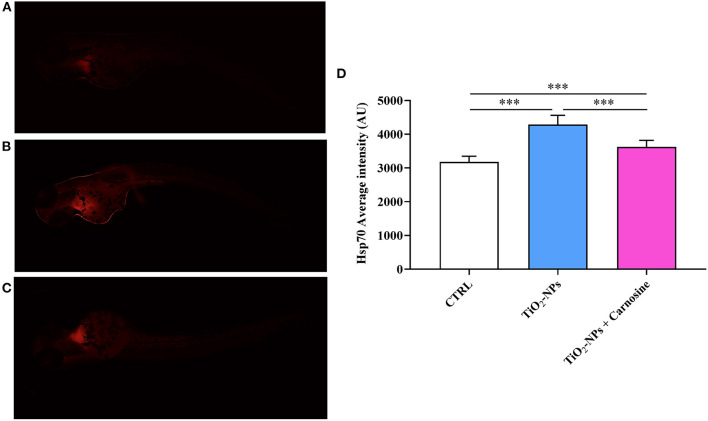
Hsp70 expression levels in **(A)** untreated larvae, **(B)** TiO_2_-NPs-treated larvae, and **(C)** TiO_2_-NPs-treated larvae in the presence of carnosine 20 mM (1 h pre-treatment). Carnosine was kept during the exposure to TiO2-NPs. The average fluorescence intensity (AU) of at least 5 values for fixed larva is reported in **(D)**. SD is represented by vertical bars. ***Significantly different, *p* < 0.001.

**Figure 7:**
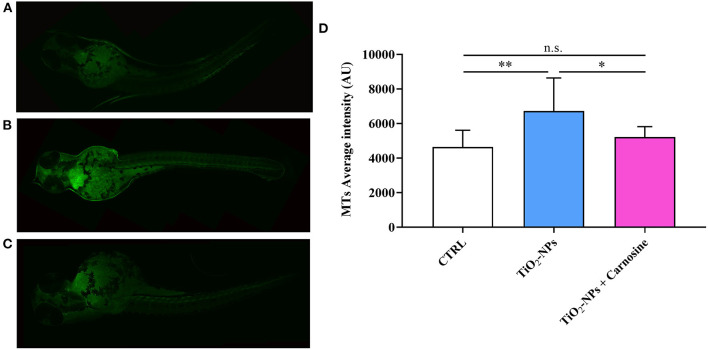
MTs expression levels in **(A)** untreated larvae, **(B)** TiO_2_-NPs-treated larvae, and **(C)** TiO_2_-NPs-treated larvae in the presence of carnosine 20 mM (1 h pre-treatment). Carnosine was kept during the exposure to TiO2-NPs. The average fluorescence intensity (AU) of at least 4 values for fixed larva is reported in **(D)**. SD is represented by vertical bars. *Significantly different, *p* < 0.05; **Significantly different, *p* < 0.01; n.s., not significant.

Immunohistochemical analysis suggests the ability of TiO_2_-NPs to induce Hsp70 protein expression in the yolk sac and at the beginning of the tail (*p* < 0.001 vs. CTRL and *p* < 0.001 vs. TiO_2_-NPs + Carnosine) (Figure 6). Of note, despite the ability of carnosine to significantly decrease the inductive effect of TiO_2_-NPs, a value still higher compared to untreated larvae was measured. A similar trend was observed when analyzing MTs expression levels. As depicted in Figure 7, the exposure of larvae to TiO_2_-NPs led to a significant (*p* < 0.01) increase in MTs expression levels compared to untreated larvae. Again, the pre-treatment of larvae with carnosine led to MTs expression levels significantly lower (*p* < 0.05) if compared larvae exposed to TiO_2_-NPs only. It is worth mentioning that carnosine treatment led to fluorescence vales comparable to that measured in untreated larvae, representing the negative controls.

## 4. Discussion

Oxidative stress is due to an unbalance occurring between the production of ROS and reactive nitrogen species (RNS) and the diminished cellular antioxidant defenses (55, 56). It is well-known that these reactive species, under basal/physiological conditions, are involved in different cellular processes such as cellular homeostasis and metabolism, signaling and redox state, being also extremely important in the immune response and pathogen clearance (57). On the other hand, the excess of ROS/RNS production can lead to the damage of fundamental macromolecules including DNA and proteins and has been linked to the development of various diseases such as cancer, cardiovascular disease, diabetes, PD, and AD (58–61).

ROS can have an adverse impact on the embryo and fetal development (62) and also play a key role in the pathophysiology of different adverse effects, from cardiotoxicity to neurotoxicity (63). Therefore, current studies in molecular toxicology and nanotoxicology are directed to better identify the preclinical toxicity of new drugs and/or NPs in order to prevent toxicity in humans. Recent studies with zebrafish focused on new drug discovery toxicology suggest that this tiny vertebrate represents the ideal tool to better understand disease biology and drug toxicity (44, 64). This becomes particularly relevant when considering the field of NPs and their relevance for human toxicology (65).

Currently, the use of NPs in industry, biology, and medicine is attracting a lot of attention. Numerous studies have been aimed at evaluating their biosafety, toxicity, and/or possible side effects both *in vitro* and *in vivo* (66, 67). Among thousands of different NPs, metal oxide NPs, and in particular TiO_2_-NPs, have shown different applications, including the production of solar cells, food wraps, pharmaceuticals, lacquers, medical devices, gas sensing, photocatalyst, and cosmetic, just to name a few (68). Despite this enormous potential, i*n vitro* cells' studies have shown how these NPs could induce oxidative stress (69), inhibit cell cycle (70), lead to inflammatory responses (71) and dysregulated autophagy (72). *In vivo* studies have highlighted how TiO_2_-NPs-induced oxidative stress contributes to organ dysfunction (73). Additionally, as a consequence of their intensive applications, TiO_2_-NPs are often released into aquatic environments (74), increasing the exposure of humans and ecosystems to NPs (75).

According to this scenario, in the present study we first investigated the effects of carnosine or TiO_2_-NPs exposure on the development and survival of zebrafish embryos/larvae. Zebrafish, as discussed above, represents an excellent tool in drug discovery toxicology to study oxidative stress-linked disorders (45, 46, 76) and to test new drugs endowed with an antioxidant activity (77, 78), while the selection of carnosine is related to its well-known multimodal mechanism of action in neurodegenerative disorders (30), including a strong direct and indirect antioxidant activity (79). With specific regards to zebrafish development, carnosine-like peptides have been linked to olfactory and visual functions (47), while the effects of carnosine exposure during zebrafish embryonic development have not been fully elucidated.

In our study, carnosine did not alter, at none of the concentrations considered, the development of zebrafish larvae (Figure 1). The absence of toxic effects was expected since both preclinical (80) and clinical (81, 82) studies have clearly demonstrated that this dipeptide is essentially non-toxic and well-tolerated, without known drug interactions and severe adverse effects (83, 84). It is also worth mentioning that despite the absence of significant deleterious effects of chronic exposure to carnosine (0.01 μM to 10 mM) on embryonic development, treatment with 100 mM carnosine can result in developmental delay and compromised larval survival (47).

According to previous studies carried out on zebrafish showing that TiO_2_-NPs did not cause any toxicity to zebrafish embryos and larvae (85), and confirmed by our results (Figure 4), the exposure to TiO_2_-NPs did not affect the hatching rate of zebrafish embryos and did not cause malformation on the larvae, despite the well-recognized ability of these NPs to cause oxidative stress phenomena (28).

Starting from this evidence, the subsequent experiments described in the present study were purposely designed in order to determine whether a non-toxic amount of TiO_2_-NPs is capable to increase oxidative stress, measured in terms of total ROS production and protein expression of two well-known markers of cellular stress (represented by Hsp70 and MTs) in zebrafish larvae, as well as the therapeutic potential of carnosine in counteracting the pro-oxidant effects of NPs.

When evaluating the production of ROS under our experimental conditions, it was found a massive increase of these reactive species in larvae exposed to TiO_2_-NPs (Figure 5). Notably, the presence of carnosine completely abolished the pro-oxidant effects of NPs, giving ROS levels superimposable to that measured in untreated larvae. These results are in agreement with a multitude of *in vitro* and *in vivo* studies showing carnosine antioxidant activity and its preclinical potential as an antidote. In particular, the observed results could be related to the ability of carnosine to directly interact with and decrease different reactive species such as superoxide anion and hydroxyl radicals (86, 87), nitric oxide (88), cytotoxic carbonyl species (89), and aldehydes (1). Carnosine has also been able to act indirectly through the enhancement of the endogenous antioxidant machinery (90–93). As reported by Chan et al., the dietary supplementation with carnosine was able to decrease the formation of thiobarbituric acid reactive substances in rats (94). The ability of carnosine to counteract oxidative stress has also been demonstrated in astrocytes, oligodendrocytes, and neuronal cultures (95–98). It is worth pointing out that recent studies have shown carnosine ability to decrease NPs-induced ROS formation in lung and microglial cells (99), while this dipeptide was able to rescue 4-hydroxynonenal (HNE)-induced retinal phenotype in *aldh3a1* zebrafish larvae mutants (31).

In the present study, we also demonstrated for the first time that the ability of carnosine to counteract oxidative stress was, at least in part, related to its modulatory activity on Hsp70 and MTs, two well-known biomarkers in translational medicine of oxidative stress response (100, 101). The enhanced Hsp70 and MTs protein expression levels were detected in zebrafish larvae following the treatment with TiO_2_-NPs (Figures 6, 7), in accordance with previous studies showing the increased expression of these markers after the exposure to metals (102–104), or other pro-oxidant/pro-inflammatory stimuli such as ionizing radiation (105), intracellular β-amyloid (106), lipopolysaccharides (107), and interferons (108). Notably, Hsp70 and MTs protein expression levels were down-regulated by the administration of carnosine to zebrafish larvae. This negative regulation of Hsp70 and MTs could be attributable to an indirect activity of carnosine able to decrease ROS and then oxidative stress, an event strictly connected to the over-expression of these proteins (17, 109–111) and that can significantly contribute to the preclinical efficacy and the therapeutic potential of carnosine as an antidote in human toxicology.

## 5. Conclusion

In the present study we demonstrated that carnosine, when used at physiological concentrations ranging from 0.1 to 20 mM, does not alter the phenotypes of both embryos and larvae of zebrafish at different hpf in terms of coagulated embryos, lack of somite formation, non-detachment of the tail, and lack of heartbeat. When tested in a well-known oxidative stress model represented by zebrafish larvae challenged with TiO_2_-NPs, carnosine significantly decreased ROS levels and this antioxidant activity was paralleled by its ability to rescue the protein expression levels of two well-recognized oxidative stress response markers represented by Hsp70 and MTs. Our results suggest a therapeutic potential of carnosine as a new pharmacological tool in the context of pathologies characterized by oxidative stress such as PD and AD.

## Data availability statement

The original contributions presented in the study are included in the article/Supplementary material, further inquiries can be directed to the corresponding author.

## Ethics statement

Ethical review and approval were not required for the animal study because no one authorization is required for experiments with larvae, before yolk sac resorption.

## Author contributions

Conceptualization/design of the study and supervision: GC and MB. Methodology: ES, RP, and ET. Software analysis: GC, ES, RP, VC, and AP. Validation, resources, and funding acquisition: GC, FCar, and MB. Formal analysis and investigation: GC, ES, RP, VC, AP, ET, and FCap. Data curation: GC, RJ, and AS. Writing—original draft preparation: GC, ES, VC, and AP. Writing, review, and editing: GC, ES, RP, VC, AP, ET, FCap, RJ, AS, FCar, and MB. Visualization: GC, RJ, AS, FCar, and MB. Project administration: GC. All authors have read and agreed to the submitted version of the manuscript.

## Supplementary material

Supplementary Video 1 CTRL 48 hpf.

Supplementary Video 2 CTRL 72 hpf.

Supplementary Video 3 Car 0.1 mM 48 hpf.

Supplementary Video 4 Car 0.1 mM 72 hpf.

Supplementary Video 5 Car 1 mM 48 hpf.

Supplementary Video 6 Car 1 mM 72 hpf.

Supplementary Video 7 Car 10 mM 48 hpf.

Supplementary Video 8 Car 10 mM 72 hpf.

Supplementary Video 9 Car 20 mM 48 hpf.

Supplementary Video 10 Car 20 mM 72 hpf.
